# Structural and biophysical characterization of the tandem substrate-binding domains of the ABC importer GlnPQ

**DOI:** 10.1098/rsob.200406

**Published:** 2021-04-07

**Authors:** Evelyn Ploetz, Gea K. Schuurman-Wolters, Niels Zijlstra, Amarins W. Jager, Douglas A. Griffith, Albert Guskov, Giorgos Gouridis, Bert Poolman, Thorben Cordes

**Affiliations:** ^1^ Molecular Microscopy Research Group, Zernike Institute for Advanced Materials, University of Groningen, Nijenborgh 4, 9747 AG Groningen, The Netherlands; ^2^ Groningen Biomolecular Science and Biotechnology Institute, Zernike Institute for Advanced Materials, University of Groningen, Nijenborgh 4, 9747 AG Groningen, The Netherlands; ^3^ Department of Chemistry, Center for Nanosciences (CeNS) and Center for Integrated Proteins Science Munich (CiPSM), Ludwig Maximilians-Universität München, Butenandtstraße 11, 81377 Munich, Germany; ^4^ Physical and Synthetic Biology, Faculty of Biology, Großhaderner Straße 2-4, Ludwig-Maximilians-Universität München, 82152 Planegg-Martinsried, Germany; ^5^ Moscow Institute of Physics and Technology (MIPT), Institutskiy Pereulok 9, Dolgoprudny, Moscow Region 141701, Russian Federation; ^6^ Structural Biology Division, Institute of Molecular Biology and Biotechnology (IMBB-FORTH), Nikolaou Plastira 100, Heraklion, Crete, Greece

**Keywords:** ABC transporter, substrate-binding protein, Förster resonance energy transfer, protein-induced fluorescence enhancement, single-molecule spectroscopy, tandem substrate-binding domains

## Abstract

The ATP-binding cassette transporter GlnPQ is an essential uptake system that transports glutamine, glutamic acid and asparagine in Gram-positive bacteria. It features two extra-cytoplasmic substrate-binding domains (SBDs) that are linked in tandem to the transmembrane domain of the transporter. The two SBDs differ in their ligand specificities, binding affinities and their distance to the transmembrane domain. Here, we elucidate the effects of the tandem arrangement of the domains on the biochemical, biophysical and structural properties of the protein. For this, we determined the crystal structure of the ligand-free tandem SBD1-2 protein from *Lactococcus lactis* in the absence of the transporter and compared the tandem to the isolated SBDs. We also used isothermal titration calorimetry to determine the ligand-binding affinity of the SBDs and single-molecule Förster resonance energy transfer (smFRET) to relate ligand binding to conformational changes in each of the domains of the tandem. We show that substrate binding and conformational changes are not notably affected by the presence of the adjoining domain in the wild-type protein, and changes only occur when the linker between the domains is shortened. In a proof-of-concept experiment, we combine smFRET with protein-induced fluorescence enhancement (PIFE–FRET) and show that a decrease in SBD linker length is observed as a linear increase in donor-brightness for SBD2 while we can still monitor the conformational states (open/closed) of SBD1. These results demonstrate the feasibility of PIFE–FRET to monitor protein–protein interactions and conformational states simultaneously.

## Graphical abstract

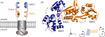

## 1. Introduction

**Figure 1.**
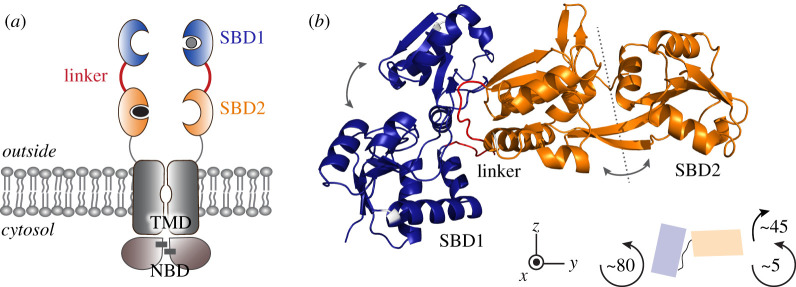
Domain organization of GlnPQ. (*a*) Schematic depiction of GlnPQ. The homodimer is composed of two subunits: GlnP comprising the TMD linked to SBD2 and SBD1 and GlnQ (NBDs). SBD1 and SBD2 are shown in blue and orange, respectively. Substrates are depicted in grey (asparagine) and black (glutamine). Black bars in the NBDs indicate two molecules of ATP. (*b*) Crystal structure of the tandem SBD1-2 with the same colouring scheme as in (*a*). Both SBDs capture amino acids between their two lobes by closing perpendicularly to their hinge region (grey dashed line; grey arrows). The linker region (red) is close to the hinge region of SBD1. Both SBDs are oriented such that SBD2 appears rotated by approximately 75° and 45° along the *x*- and *y*-axis, respectively. For simplicity, only one of the two orientations observed in the crystal structure (chain A) of the SBDs in the tandems is shown.

ATP-binding cassette (ABC) transporters represent a major family of transmembrane proteins, involved in a variety of cellular processes [1–3], including nutrient uptake, antibiotic and drug-resistance, lipid trafficking and cell volume regulation. They mediate uphill transport of solutes across cellular or organellar membranes using hydrolysis of cytosolic ATP. The core of an ABC transport system is composed of two transmembrane domains (TMDs) and two highly conserved nucleotide-binding domains (NBDs) [4] (figure 1*a*). In bacterial ABC importers, additional substrate-binding proteins (SBPs) or domains (SBDs) specifically capture and deliver substrates to the TMDs for transport [5,6]. In some cases, multiple distinct SBPs enable the transport of distinct substrates via the same translocator domain [7–10].

The ABC import systems are divided into three categories according to the overall structure of their TMDs, which dictates the mechanism by which they facilitate transport [11–13]. Type I and II ABC importers make use of extra-cytoplasmic SBPs or SBDs that capture ligands directly from the surrounding medium. Substrate uptake is then a multistep process: after binding of the ligand to the SBP, the latter docks onto the TMDs and releases the substrate into a pocket in the translocation pathway. Uphill substrate transport is facilitated by alternating access of the pocket to the opposing sides of the membrane. Type III import systems [14–16] feature a membrane-integrated SBP called *S* factor. The SBPs of Type I/II ABC importers in Gram-negative bacteria are found in the periplasm, where they freely diffuse to capture their substrates. In Gram-positive bacteria, archaea and some Gram-negative bacteria, the SBPs are directly linked to the membrane by a lipid anchor or are tethered to the translocator (hence the name SBD).

In this contribution, we focus on the Type I ABC importer GlnPQ, which features two distinct SBDs in tandem: SBD1 and SBD2 (figure 1*a*). This multi-subunit protein is an essential uptake system for glutamine and/or glutamic acid in a variety of non-pathogenic (e.g. *Lactococcus lactis*) and pathogenic Gram-positive bacteria (e.g. *Streptococcus pyogenes, Staphylococcus aureus*, *Enterococcus faecalis*). The system can also transport a variety of non-essential amino acids [7–10]. GlnPQ is a homodimer composed of two subunits: GlnP and GlnQ. GlnP comprises the TMDs, which are C-terminally linked to SBD2 and SBD1, whereas GlnQ is the cytosolic NBD. SBD2 is attached to the TMD via a 19 amino acid long flexible linker (figure 1*a*; grey) and connected to SBD1 via a 14-amino-acid-long linker (figure 1*a*, red) [17]. This arrangement facilitates the fast delivery of ligands from the SBDs to the translocator. GlnPQ imports glutamine, glutamic acid and asparagine [8]. Whereas the proximal SBD2 exclusively binds glutamine with a *K*_D_ of approximately 0.9 µM, the distal SBD1 binds both: asparagine with high affinity (*K*_D_ = 200 nM) and glutamine with a low affinity (*K*_D_ = 90 µM) [8]. The SBDs have thus evolved distinct substrate specificity.

Both crystallography and single-molecule Förster resonance energy transfer (smFRET) experiments on the single SBD1 and SBD2 showed that substrate binding is linked to a conformational change of the corresponding SBD from an open *apo* conformation to a closed liganded conformation [18–21]. The results also implied an induced-fit-type ligand-binding mechanism, where conformational dynamics are induced by ligand–SBD interactions similar to later demonstrated for other SBPs [22–26]. Additionally, it was shown that the opening of the SBDs and ligand release can be one rate-limiting step in the transport cycle and that the closed conformation triggers ATP-hydrolysis and transport [18]. More recently, it was shown that some SBPs and SBDs can recognize multiple distinct ligands and that the ligand–SBP or SBD complexes formed do not necessarily share a single translocation competent conformation [19]. Instead, transport specificity was determined by the formation of conformers capable of allosteric coupling with the translocator, while retaining conformational dynamics permissive for ligand release. These recent findings provide a new understanding of the mechanistic diversity that enables ABC importers to achieve substrate selectivity [19,27].

A particularly interesting feature of the GlnPQ importer is the presence of the two SBDs fused in tandem to the TMD, generating four substrate-binding sites close to the translocation pathway and SBD competition for docking onto the translocator. Even though the mechanism of ligand binding for the individual SBDs is well characterized, it is not clear how interactions between the SBDs might affect transport. Although we could recently show that changes in the inter-domain distances can affect transport and ATPase activity [17,28], what this reveals about the native transport mechanism is as yet not fully clear. Also, possible domain interactions or functional cooperativity between the SBDs in the tandem still must be assessed. The key questions are whether the properties of the single SBDs are the same as when they are present in the tandem and whether there is evidence for functional cooperativity (e.g. that binding of substrate to one SBD alters ligand binding or conformational dynamics of the other).

In this work, we therefore focus on studying the structural and biochemical consequences of connecting two SBDs by a flexible linker. We present crystallographic, biochemical and biophysical data. We first determined the crystal structure of the SBD-tandem in its ligand-free form and used smFRET-based spectroscopy to determine the underlying conformations and substrate-binding affinities of the individual SBDs within the tandem to disentangle the contributions of the individual domains. We find that tandem ligand-binding domains have identical structures as compared to isolated SBDs and both domains operate largely independently of each other in the tandem. The ligand binding was only affected marginally by the adjoined domains for extremely short artificial linkers. This finding raises the question about how optimized the length of the linker connecting the two SBDs in GlnPQ is and whether cooperativity can be induced by changing this length. To elucidate the interaction of the two domains and the flexibility provided by the connecting linker in the tandem, we employed inter-domain FRET and explored the suitability of our recently introduced PIFE–FRET assay [29,30], which combines smFRET with protein-induced fluorescence enhancement (PIFE) for the study of protein–protein interactions.

## 2. Results

### 2.1. Crystallization and structure determination

We solved the crystal structure of the unliganded tandem SBD1-2 domain at 2.8 Å resolution (PDB ID 6H30; see figure 1*b*; electronic supplementary material, figure S1 and table S1). Crystals of the unliganded tandem SBD1-2 in buffer supplemented with MES were grown with the hanging drop vapour diffusion method. The crystals belonged to the C222_1_ space group and contained two polypeptide chains per asymmetric unit with 58% solvent content (electronic supplementary material, figure S1A). Each of the two chains comprises two SBDs linked via a 14-amino-acid loop. The individual SBDs consist of two α/β subdomains. In SBD1, the large α-domain comprises residues 29–113 and 207–251, while the small β-domain is made up of residues 114–206 (see figure 1*b*, blue domain). The large α-domain in SBD2 is formed by residues 255–345, and residues 346–440 are of the small β-domain (see figure 1*b*, orange domain). Both domains in SBD1 and SBD2 are connected by two anti-parallel β-strands, a common feature in SBPs. The two SBDs are structurally classified in the sub-cluster F-IV [5]. The binding site for the substrates is localized between the two domains.

### 2.2. Structural comparison of single and tandem SBDs

The crystallized tandem SBD1-2 structure reveals MES molecules in the binding pockets of the open state (electronic supplementary material, figure S1). An asymmetric unit contains two SBD1-2 monomers that are oriented head to tail (electronic supplementary material, figure S1A). We found that the SBDs in the tandem have identical structures to those of the individual SBDs as revealed by the superposition of SBD1-2 structure with those for unliganded SBD1 (PDB ID 4LA9, rmsd of 0.5 Å) and SBD2 (PDB ID 4KR5, rmsd of 1.1 Å) (electronic supplementary material, figure S1B; table S1). The linker sequence (depicted in red in figure 1 and electronic supplementary material, S1) connects the last α-helix of SBD1 to the first β-sheet of SBD2 and comprises the residues Gly-248 to Val-261. In comparison to other homologues, this sequence is very short [8,17]: close homologues of SBD1-2 found in *Streptococcus pneumoniae* and *Enterococcus faecalis* show an extra insertion in this region of 11 amino acids making the linker almost twice as long. We speculate that the connecting linker between the domains should still provide some flexibility as suggested from the way molecules are packed within the crystal (electronic supplementary material, figure S1C). Both domains of the tandem SBD1-2 are oriented differently within the crystal unit cell: the superposition along SBD1 of both domains in tandem SBD1-2 reveals a rotation of approximately 45° for SBD2 with close contact to the hinge region of SBD1. For simplicity, only one of the two orientations observed in the crystal structure (chain A) is shown in figure 1*b*.

### 2.3. SBD substrate affinity and specificity

**Figure 2.**
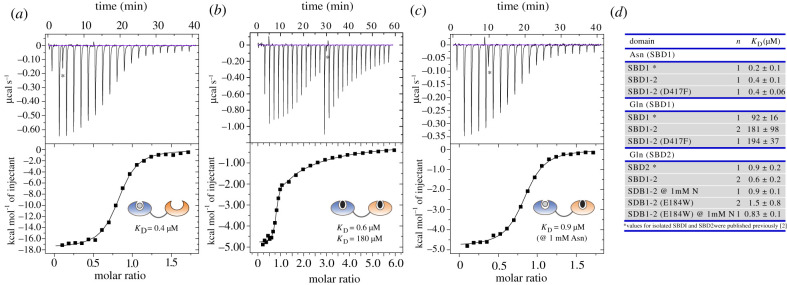
Ligand-binding affinities of tandem SDB1-2 as determined by ITC. (*a*) Binding of asparagine to SBD1-2 occurs to SBD1, shown in blue. (*b*) In the absence of asparagine, glutamine binds to both domains in the tandem with *K*_D_ values of 180 µM and 0.6 µM for SBD1 and SBD2, respectively. (*c*) SBD2 binds glutamine with a *K*_D_ of 0.9 µM, which was determined by blocking SBD1 with a high concentration (1 mM) of asparagine. Spikes due to leakage of the syringe (marked by *) have been excluded from the analysis. (*d*) Overview of *K*_D_ values that are summarized in electronic supplementary material, table S2. The *K*_D_ values were obtained from five biological replicates. Fit values for *K*_D_ are based on the data shown in the figure.

We next analysed the binding properties of the tandem SBD1-2 in comparison to the published ones from single SBD1 and SBD2, using isothermal titration calorimetry (ITC). Figure 2 shows the binding curves for the two high-affinity ligands of SBD1-2 (see electronic supplementary material, table S2 for details). SBD1 within the tandem SBD1-2 binds asparagine with a dissociation constant *K*_D_ of 400 ± 100 nM (figure 2*a*), which is similar to isolated SBD1 (*K*_D_ = 200 ± 100 nM [8]). The titration of SBD1-2 with glutamine reveals two binding sites, one with high and one with low affinity since glutamine can be bound by both SBDs (figure 2*b*). The *K*_D_ values for binding of glutamine were 0.6 ± 0.2 µM (for SBD2) and 180 ± 100 µM (for SBD1), which are similar to the values observed for the isolated SBDs (electronic supplementary material, table S2) [8]. In the presence of saturating concentration of asparagine, the *K*_D_ for binding of glutamine to SBD2 in the tandem and the single domain is the same (figure 2*c*). We can thus conclude that the proximity of the domains in the tandem, which is enforced by the linker does not alter ligand affinities.

This conclusion is further supported by experiments on mutants with one inactive and one functional ligand-binding domain (figure 2*d*). It was shown previously that isolated variants SBD1(E184 W) and SBD2(D417F) do not bind ligand and remain in the open conformation even in the presence of substrates [18,31]. ITC experiments on similar tandem variants SBD1(E184 W)-2 (inactive SBD1) and SBD1-2(D417F) (inactive SBD2) show that the functional SBD of the tandem is not affected by the inactivation of the other SBD (electronic supplementary material, figure S2 and table S2). When comparing SBD1(E184 W)-2 to the wild-type SBD1-2 (electronic supplementary material, figure S2A), we can conclude that glutamine-induced conformational fluctuations in SBD1 do not affect the binding of glutamine to SBD2. Similarly, experiments with SBD1-2(D417F) (electronic supplementary material, figure S2B,C) imply that the binding of a ligand to the SBD2 does not affect binding to SBD1. In accordance, the binding isotherms of SBD1-2 for glutamine are a superimposition of those of SDB1 plus SBD2.

### 2.4. Binding affinities and states of single and tandem SBDs probed by smFRET

**Figure 3.**
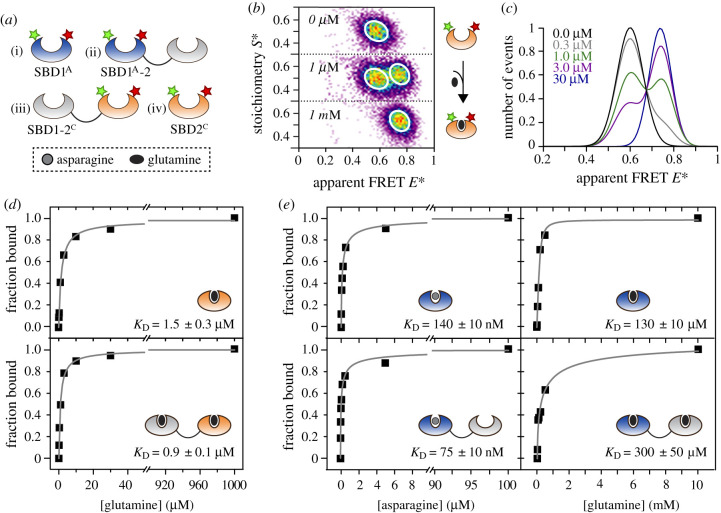
ALEX spectroscopy on single and tandem SBDs in GlnPQ. (*a*) Design of smFRET assay to monitor intramolecular SBD conformational states. (*b*) Confocal based ALEX spectroscopy of SBD2^C^ labelled with Alexa Fluor 555 and Alexa Fluor 647 maleimide in the presence of 0 µM, 1 µM and 1 mM of glutamine. SBD2^C^ shows an apparent FRET value of 0.58 in the unliganded state. The apparent FRET E* shifts to 0.74 under saturating concentrations of glutamine. (*c*) Apparent FRET E* histograms as a function of varying ligand concentration. The addition of glutamine to SBD2^C^ shifted the population of molecules from a low (unliganded) to a high FRET state (closed liganded). At concentrations close to the *K*_D_ both populations were similar in occurrence. (*d*,*e*) Binding affinity determination of single and tandem SBDs by ALEX spectroscopy. (*d*) SBD2^C^ as single domain and tandem SBD1-2^C^ gave *K*_D_ values for glutamine of 1.5 and 0.9 µM, respectively. The ratio of populations is defined as the ratio of areas in the case of the closed liganded population versus (unliganded and closed liganded population combined). (*e*) SBD1^A^ as single domain and tandem SBD1^A^-2 gave an apparent *K*_D_ for asparagine of 140 and 75 nM, respectively; the corresponding values for glutamine are 130 and 300 µM. Errors indicated were obtained directly from the fit in the respective dataset.

In addition to our ITC experiments, we also used smFRET assays as an independent approach to examine whether there is functional cooperativity between the domains in the tandem. We employed smFRET [32–35] in a fashion similar to previous work [18,19] to monitor the conformational state changes and to simultaneously extract the substrate-binding affinity of the individual domains within the tandem. In the smFRET assay (figure 3*a*), we observe conformational changes directly as differences in the FRET efficiency, where the open conformation is characterized by a low FRET state and the closed substrate-bound conformation has a higher FRET state (figure 3*b*). We employed different cysteine variants as described previously [18,19] and created the corresponding variants for the tandem SBD1-2 (figure 3*a*). Cysteine residues were located at G87C and T159C in SBD1 of the tandem SBD1-2 (figure 3*a*(i,ii) and T369C and S451C in SBD2 of SBD1-2 (figure 3*a*(iii,iv)). We denote the two cysteine-backgrounds as subscripts on the single SBDs, such as SBD1^A^-2 and SBD1-2^C^ (a summary of the short notations of all proteins including mutations is provided in electronic supplementary material, table S3). The occurrence of potentially problematic fluorophore–protein interactions was ruled out by steady-state anisotropy experiments (electronic supplementary material, table S4).

**Figure 4.**
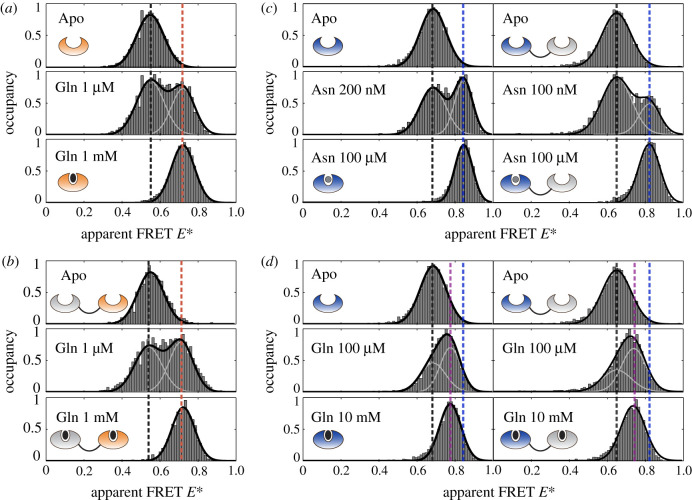
Conformational states of isolated and tandem SBDs probed by ALEX spectroscopy. (*a*,*b*) Single and tandem-linked SBD2^C^ in the presence of glutamine. Both proteins are characterized by two FRET states: the apo-state at 0.58 (black line) and the closed liganded state at 0.74 (orange line). (*c*,*d*) Single and tandem-linked SBD1^A^ mutant in presence of different ligands. Both proteins show a low FRET value of approximately 0.65 in the apo-state (black line). (*c*) Upon addition of asparagine: both proteins start closing, which is observed as an additional high FRET state at approximately 0.82 (blue line). At the *K*_D_, two distinct populations are observed at an equal ratio. For saturating concentrations of asparagine, both mutants are fully closed. (*d*) In the presence of glutamine, a gradual shift in FRET is observed hinting towards fast interconversion of states. For saturating concentration, both mutants show an intermediate FRET state of approximately 0.74 (purple line), which is lower than in the case of asparagine (blue line).

We examined ligand binding by stepwise addition of substrate to a very dilute protein solution (≈50 pM) and monitored the conformational transition between the open unliganded and the closed liganded state, which is manifested as a change in FRET efficiency (figure 3*b*). We employed µs-alternating laser excitation (ALEX) spectroscopy [36–38] with alternating laser excitation at 532 and 640 nm, where fluorescently labelled biomolecules diffuse through the excitation volume of a confocal microscope. After stochastic labelling of SBD2^C^ and the tandem SDB1-2^C^ with Alexa Fluor 555- and Alexa Fluor 647-maleimide, a single population was observed that was distributed around an apparent FRET efficiency E* of 0.58 (figure 3*b*; figure 4*a*,*b*; electronic supplementary material, figure S3 and tables S5, S6). Under saturating concentrations of glutamine, the apparent FRET E* value shifted to 0.74 for both proteins (electronic supplementary material, figure S3), supporting the idea that both proteins undergo identical conformational changes. Sorting the molecules at the given substrate concentrations according to their FRET value (figure 3*c*) revealed that the amplitude of the *apo*-protein gradually decreased with an increasing concentration of ligand, while the closed, liganded state at 0.74 increased in parallel. We obtained a binding curve from the ratio of the number of molecules in the closed liganded state over the total number of recorded molecules (figure 3*d*). It yielded an apparent *K*_D_ of approximately 0.9 µM for SBD1-2^C^ and approximately 1.5 µM for SBD2^C^, which is consistent with ITC experiments (figure 2; electronic supplementary material, table S2). From this, we can conclude that glutamine binding to SBD2 was unaffected by the presence of SBD1 and ligand binding correlates with conformational changes in both isolated SBD2 and the tandem.

By contrast to SBD2, SBD1 binds both asparagine and glutamine (figure 3*e*; electronic supplementary material, figures S4 and S5). For smFRET experiments, SBD1 was labelled at G87C and T159C with Alexa Fluor 555 and Alexa Fluor 647 maleimide. The variant showed a single population with an apparent FRET of 0.64 in the *apo*-state (figure 4*c*). In the presence of asparagine and glutamine, SBD1^A^-2 undergoes a conformational transition to the same closed liganded state as for isolated SBD1^A^ (figure 4*c*,*d*). The apparent *K*_D_ of 75 nM for asparagine binding to SBD1^A^-2 (figure 3*e*, left) is similar to that obtained for isolated SBD1 (*K*_D_ = 140 nM). The respective *K*_D_ values for glutamine binding are also similar with values of 300 µM for tandem SBD1^A^-2 and 130 µM for SBD1^A^.

We conclude from a combined inspection of the ITC and smFRET experiments that (i) the ligand dissociation constants of single and tandem SBDs are not notably different. (ii) Isolated SBDs and their tandem counterparts show identical conformational states in the presence and absence of their ligands (i.e. both high- and low-affinity ligands trigger the formation of similar ligand-bound closed states).

### 2.5. Domain orientation of SBD1 and SBD2 in the tandem in solution

**Figure 5.**
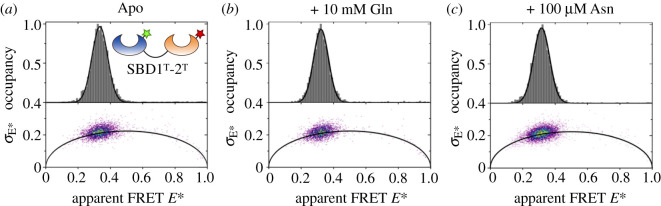
µs-ALEX Spectroscopy and burst variance analysis to probe the inter-domain distance and dynamics in the tandem SBD1^T^-2^T^. Top: FRET histograms of the tandem SBD1^T^-2^T^ labelled with Alexa Fluor 555- and Alexa Fluor 647-maleimide, in the (*a*) apo-state and under saturating concentrations of (*b*) glutamine and (*c*) asparagine. The protein shows a low FRET value of 0.33 in the apo-state, which does not change upon the addition of ligands; also, the width of the distribution does not change. Double-labelled protein species were identified in the *ES*-histograms using a stoichiometry range of *S* = 0.25–0.6.

To examine whether the flexible linker allows domain re-orientation within the tandem, and to determine the functional relevance of the orientation of the domains in the crystal structure, we employed an inter-domain single-molecule FRET assay. For this, we designed a double cysteine variant with one fluorophore anchor point in SBD1 (A136C) and one in SBD2 (T369C), which we denoted as SBD1^T^-2^T^. As a structural reference, we selected chain A from the crystal structure which suggests an approximately 65 Å inter-probe distance for the variant considering the *C*_β_ distances of the respective amino acids. We then labelled the variant with Alexa Fluor 555 and Alexa Fluor 647 (figure 5*a*). The resulting smFRET histogram in the absence or presence of ligand shows a single population at low apparent FRET efficiency around 0.33 (figure 5*a*).

In addition to the centre position of the FRET distribution, its width also reports on the underlying dynamics in the system. By contrast to the molecules in static samples where any broadening of the peak would exclusively originate from shot-noise, additional fast conformational transitions of the molecules in dynamic samples, on the order of the diffusion time, will result in an additional broadening [39]. A burst variance analysis (BVA) on the µs-ALEX data of SBD1^T^-2^T^ tandem shows that the width of the population with 0.046 hardly deviates from the theoretical shot-noise limit of 0.037, which was determined using the mean number of photons per burst, which was similar for each condition (figure 5). These small differences in width imply that the two large domains do not re-arrange their position, or the process is much faster compared to the transit time through the confocal volume. The latter seems more likely since protein rotation should occur on time scales of 10–100 ns (considering the masses of SBD1/2), which is sufficient to allow both SBDs to adopt all relative possible orientations using the linker region as a flexible element. Moreover, the addition of saturating ligand concentrations does not change the position of the peak and thus the distance between the domains, nor the width of the peaks (figure 5*b*,*c*). It is clear from this dataset that smFRET will only allow further investigations when combined with pulsed interleaved excitation and multiparameter fluorescence detection (PIE-MFD) measurements that allow the protein system to be probed on the micro- to nanosecond time scale [40].

### 2.6. Design and biochemical characterization of linker mutants

**Figure 6.**
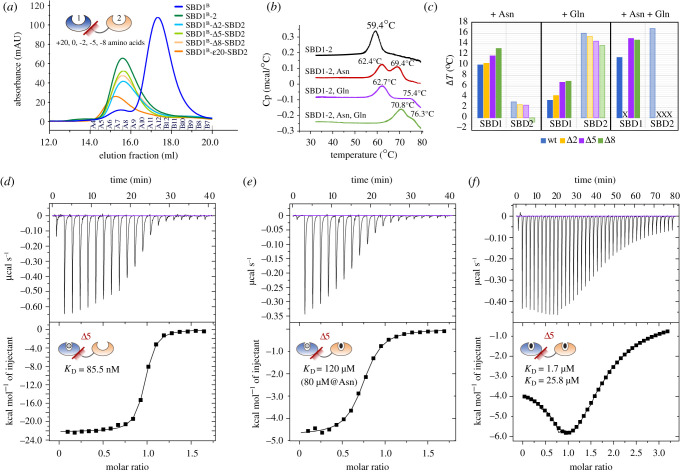
Effects of variations in linker length on the hydrodynamic radius, stability and ligand binding activity of tandem SBD1-2. (*a*) Size-exclusion of unlabelled and cysteine-containing variants on a Superdex 200 10/300 GL column. Tandem SBDs eluted around 15.5 ml and were clearly discernible from the single SBDs, which eluted around 17.5 ml. (*b*,*c*) Absolute and relative thermostability of SBD1-2 wild-type protein as measured by DSC. (*d*–*f*) ITC titrations for SBD1-Δ5-SBD2 with (*d*) asparagine, (*e*) glutamine with saturating amounts of asparagine and (*f*) glutamine.

To alter the short-distance interactions between both SBDs within the tandem SBD1-2 and to elucidate the effect of the linker length on the properties (ligand affinity and conformational states) of the tandem, we designed a range of SBD1-2 tandems with different linker lengths connecting both SBDs (figure 6; electronic supplementary material, S6).

The tandem mutants have deletions of 2, 5 and 8 amino acids within the linker at the C-terminus of Ala-251, and the deletions were made in the cysteine-backgrounds SBD1(A136C/S221C)-SBD2 and SBD1-SBD2(T369C/S451C); see electronic supplementary material, figure S6A. We denote them as SBD1^(B)^-Δ#-SBD2 and SBD1-Δ#-SBD2^(C)^, wherein # indicates the number of amino acids that are deleted from the linker sequence (electronic supplementary material, figure S6B). The superscripts refer to the cysteine-background of the mutants. We additionally produced two mutants with an extended linker of 20-amino acid, inserted between Ala-251 and Thr-252. They are named SBD1^B^-ε20-SBD2 and SBD1-ε20-SBD2^C^. A summary of the short notations for all mutants can be found in electronic supplementary material, table S3. The linker modifications did not alter the apparent hydrodynamic radius of the SBDs compared to the wild-type protein as determined by size-exclusion chromatography (figure 6*a*), except for SBD1^B^-ε20-SBD2.

Furthermore, we verified the stability of the proteins using differential scanning calorimetry (DSC) by analysing thermostability and potential differences in folding of wild-type SBD1-2 and linker mutants (figure 6*b*,*c*; electronic supplementary material, table S7). In the unliganded state, the melting temperature of both single SBDs was 59°C [41], which is the same for all tandem SBD1-2 mutants. The addition of either asparagine or glutamine increased the protein stability of the tandem resulting in a higher melting temperature of approximately 62°C for SBD1 and greater than or equal to 69°C for SBD2. SBD1 and SBD2 are unfolding separately as seen by a double peak in figure 6*b*. We assign the peak shifts to a specific SBD by comparing the melting temperatures of the tandem SBD1-2 to that of the single SBDs [41]. Figure 6*c* shows the relative thermostability of both domains for all tandem linker mutants in the presence of ligand. We find that linker deletions of 5 and 8 amino acids increase the stability of the proteins by 2–3°C. Maximal stabilization of SBD2 in SBD1-2 tandems is observed in the presence of glutamine, which was increased further in the presence of asparagine (figure 6*c*). These data suggest that the SBDs are stabilized by direct protein–protein interactions that do not occur when the linker is too long.

To characterize their biochemical properties, we analysed the linker deletion mutants by ITC and determined the thermodynamic parameters of ligand binding (Δ*H*, *T*Δ*S*, Δ*G* and *K*_D_; see electronic supplementary material, table S2). Wild-type SBD1-2 has a single binding site for asparagine and two binding sites for glutamine. The measurements with SBD1-ε20-SBD2 and SBD1-Δ2-SBD2 corroborate our observations that there is no apparent cooperativity in the binding of amino acids by the presence of the adjoining SBD with long linkers. On the other hand, the SBD1-Δ5-SBD2 mutant shows an increased binding affinity for asparagine in SBD1 (figure 6*d*), but there is only a minor effect on the binding of glutamine to SBD2. The *K*_D_ for asparagine binding in SBD1 decreases from 0.4 to 0.06 µM for SBD1-Δ5-SBD2. Also, we observe more clearly than in the wild-type protein two glutamine binding sites in SBD1-Δ5-SBD2, indicating that the *K*_D_ for glutamine binding of SBD1 is also decreased by the deletion of 5 amino (figure 6*e*,*f*) from approximately 100 to 19 µM (electronic supplementary material, table S2).

### 2.7. Simultaneous observation of inter- and intra-domain distances via PIFE–FRET

**Figure 7.**
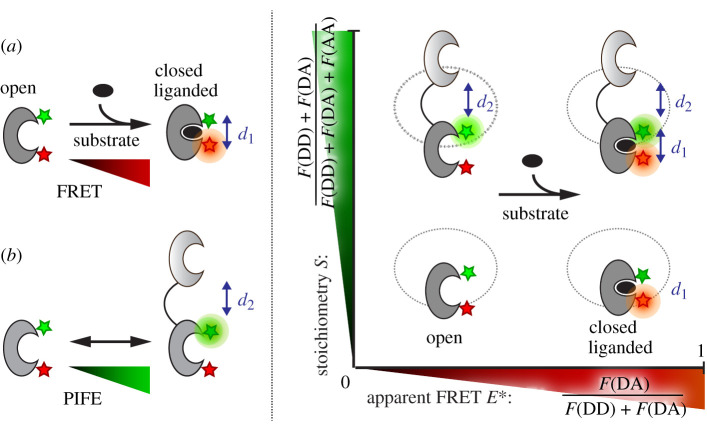
Working principle of PIFE–FRET in tandem-linked SBD1-2. (*a*) FRET between a donor and acceptor molecule reports on distance changes *d*_1_ within one of the SBDs. (*b*) PIFE can additionally report on the distance between the linked SBDs. (*c*) Readout of PIFE and FRET via ALEX spectroscopy: in a schematic *E*–*S*-histogram, conformational changes *d*_1_ within the SBD are monitored via the FRET efficiency *E*, whereas the distance *d*_2_ between the two SBDs is determined by changes in stoichiometry *S*. Both *E* and *S* are defined via photon streams in the material and methods section.

Next, a synergistic combination of smFRET (figure 7*a*) with PIFE (figure 7*b*) was used to simultaneously study inter- and intra-domain interactions in the tandem SBD1-2 (figure 7*c*). PIFE–FRET [29,30] was recently introduced by us to monitor interactions between nucleic acids and proteins concomitant to conformational changes [29,30]. A similar PIFE–FRET assay in µs-ALEX experiments has, to the best of our knowledge, not been introduced to monitor protein–protein interactions and conformational motion. In the following experiments, we aimed to probe intra-domain distance *d*_1_ via FRET, while probing the inter-domain dynamics via distance *d*_2_ using PIFE (figure 7*c*).

PIFE is based on the change of an excluded volume or in the micro-viscosity of an environmentally sensitive dye by the proximity of an adjacent protein and can be observed as a change in fluorescence brightness, lifetime or anisotropy of the dye [29,30,42]. To visualize the short distance *d*_2_ in ALEX experiments, we used stoichiometry *S*, which gives the fluorescence emission intensity coming through the donor as a percentage of the total fluorescence emission intensity. A brightness increase of the donor fluorophore due to the proximity of an adjacent protein moiety is thus observed as an increase in the stoichiometry *S* (figure 7*c*), whereas a brightness increase of the acceptor would lead to a decrease in stoichiometry. We employed the cyanine dye Cy3 as PIFE-sensor in combination with Atto647N as an environmentally insensitive FRET acceptor fluorophore. Following our theoretical framework [29,30], contributions of FRET and PIFE to the emission of the donor and acceptor can be disentangled and both the intra-domain as well as inter-domain distance can be monitored simultaneously. In the case of the tandem SBD1-2 in GlnPQ, PIFE can easily be combined with smFRET, since the assay only requires a specific set of fluorescent labels but is applicable to the same mutants as used for smFRET. The assay has thus the potential to map the interactions between both SBDs (figure 7*c*, stoichiometry axis) and the conformational states of an individual SBD (figure 7*c*, apparent FRET axis) simultaneously by a mere change of fluorophores.

### 2.8. PIFE–FRET monitors protein–protein interaction between the two SBDs

To study the interactions between the two SBDs by PIFE–FRET in further detail, we labelled one of the SBDs within the tandem with Cy3/Cy3B- and ATTO647N-maleimide. In this assay, we anticipated a brightness change of Cy3 due to the presence of the adjoining SBD (figure 7*c*). The goal was a comparison of the mean FRET and stoichiometry value of the single SBDs (SBD1^B^ and SBD2^C^), the tandem SBDs (SBD1^B^-2 and SBD1-2^C^), and the linker mutants (SBD1^B^-Δ#-SBD2 and SBD1-Δ#-SBD2^C^). Here, S-changes would be indicative of PIFE effects caused by domain–domain interactions. Labelling with Cy3B would serve as a negative control with a donor fluorophore that does not show PIFE [29,30].

**Figure 8.**
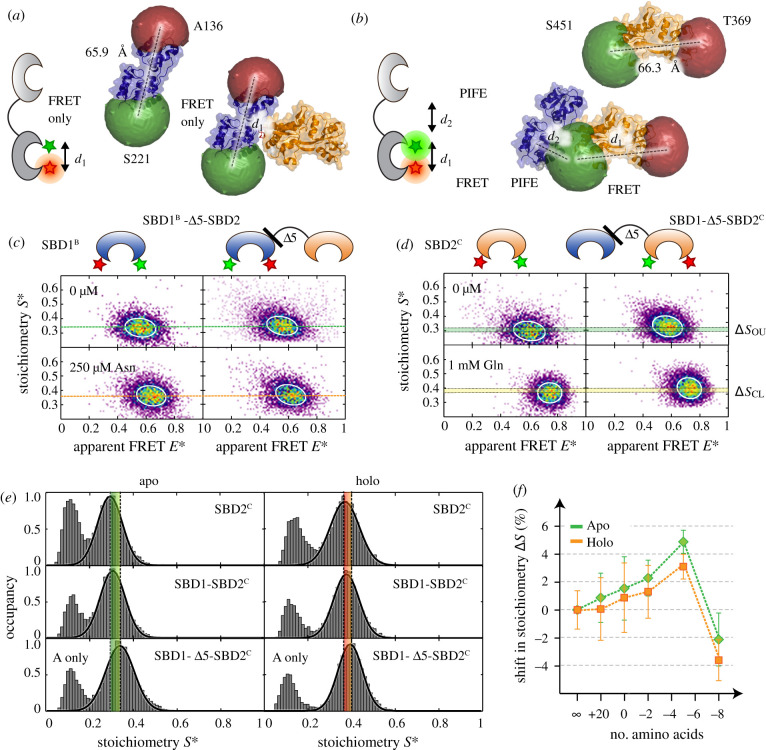
Tandem SBD1-2 investigated by PIFE–FRET. (*a*,*b*) Structure of SBD1-2, including labelling positions and accessible volumes of the dyes. (*a*) AV simulations predict no contact between the donor fluorophore on SBD1—neither at A136 nor S221—and SBD2. Note that this does not consider the case where the linker allows a flexible rotation of both domains. (*b*) In SBD2, however, the donor fluorophore at S451 is affected by the presence of SBD1, hence leading to PIFE of the fluorophore. (*c*) ALEX spectroscopy on SBD1^B^ and SBD1^B^-Δ5-SBD2 labelled with Cy3/ATTO647N in the presence and absence of asparagine. No shift in stoichiometry is observed between both mutants in an open/closed state. (*d*) ALEX spectroscopy on SBD2^C^ and SBD1-Δ5-SBD2^C^ labelled with Cy3/ATTO647N in the presence and absence of glutamine. A shift in stoichiometry is observed between SBD2^C^ and in SBD1-Δ5-SBD2^C^ for both, open unliganded and closed liganded state. (*e*) Stoichiometry histograms of SBD2^C^, SBD1-Δ5-SBD2^C^ and SBD1-Δ5-SBD2^C^ in the apo and holo-state labelled with Cy3- and Atto647N-maleimide. The stoichiometry value increases depending on the presence and distance to the second domain SBD1. (*f*) Shift in stoichiometry as a function of linker length. Mean values and standard deviations of the open (closed liganded) state represent the average of two to four independent experiments (see electronic supplementary material, table S8).

Based on the crystal structure of chain A and accessible volume (AV) calculations [43], we built a simple (and maybe oversimplified) model for AV changes caused by the absence and presence of the second SBD (figure 8*a*,*b*). Based on the models we hypothesized that for SBD1^B^-2 tandem and its linker mutants, only conformational changes *d*_1_ should be observable since no reduction of fluorophore AV is expected in the tandem (figure 8*a*). Based on the model in figure 8*b* we expect PIFE to occur when Cy3 labels position Ser-451 in SBD2 due to the steric hindrance caused by the neighbouring domain and consecutive reduction of the AV of the dye in this case (figure 8*b*). It is important to note that we perform stochastic labelling and that consequently only one of the two resulting sub-populations of labelled SBD2-proteins in the tandem (with the donor located at Ser-451) is expected to show a PIFE-signal. This fact requires careful checking of the observed effects in relation to variations of the donor–acceptor labelling ratio or if available site-specific labelling.

To test our predictions, we investigated single SBD1^B^, SBD1^B^-2 and SBD1^B^-Δ#-SBD2 variants via µs-ALEX and analysis of two-dimensional *E*/*S* histograms. The comparison of SBD1^B^ and tandem SBD1^B^-2 showed identical E* distributions with means of 0.56 and 0.63 in the apo-state and closed liganded states on the *X*-axis, respectively (figure 8*c*; electronic supplementary material, figure S7). As observed for SBD1^A^ and SBD1^A^-2, the binding affinity and conformational states in SBD1^B^-2 are not affected by the adjoining SBD2 (electronic supplementary material, figure S7 and table S5/S6). Moreover, this holds when shortening the linker between both domains, as shown for SBD1^B^-Δ5-SBD2 in electronic supplementary material, figure S7. Interestingly, there was no significant difference in stoichiometry S* for comparison of single SBD1^B^, SBD1^B^-SBD2 and SBD1^B^-Δ5-SBD2 or other linker-variants (figure 8*c*; electronic supplementary material, figure S8), which can be inspected via the mean stoichiometry of each state. This statement holds for the apo-state (figure 8*c*, green line, *S* = 0.284) and liganded holo-state (figure 8*c*, orange line, *S* = 0.290) for direct comparison of SBD1^B^ and SBD1^B^-Δ5-SBD2, an observation in line with the predictions based on the AV simulations of chain A (figure 8*a*).

Next, we characterized SBD2^C^, SBD1-2^C^ and SBD1-Δ#-SBD2^C^ mutants. Again, the single SDB2^C^ serves as a reference with one population centred at E* = 0.61 (figure 8*d*). Under saturating concentration of glutamine, the apparent FRET species shifts to 0.73. SBD2^C^ and SBD1-2^C^ show the same binding affinity and identical FRET states in the presence and absence of glutamine (electronic supplementary material, figure S9). However, the presence of the second SBD1 leads to an increase in stoichiometry from 0.291 (apo, single SBD) to 0.306 (apo, tandem SBD) and 0.339 (apo, SBD1-Δ5-SBD2^C^), also for the holo-state (figure 8*d*, green line: apo, orange line: holo).

Motivated by the small, yet significant changes, we investigated the systematics of stoichiometry changes in SBD1-Δ#-SBD2^C^ variants as a function of linker length (figure 8*e*/*f*). Starting from single SBD2, we observe a (linear) increase in stoichiometry for the tandem SBD1-SBD2^C^ reaching a maximum for SBD1-Δ5-SBD2^C^ variant and a minimum value for SBD1-ε20-SBD2^C^. These results are in line with a distance-dependent PIFE effect [29] and reveal the universal nature of PIFE as a molecular ruler, which can be applied even for protein–protein interactions as demonstrated in figure 8. To validate the interpretation of *S*-changes and the observed trends, we also performed labelling with Cy3B as a donor fluorophore, which has identical spectral properties as Cy3, but does not show PIFE effects [29] and can thus serve as the negative control. SBD1-2^C^ and SBD1-Δ#-SBD2^C^ mutants labelled with Cy3B- and ATTO647N-maleimide did not show any change in stoichiometry in any case (electronic supplementary material, figure S11). We emphasize that the shown experiments serve as a proof-of-concept that PIFE–FRET is applicable to protein–protein interactions, yet no further detailed interpretations or mechanistic conclusions could be drawn from the data.

## 3. Discussion

**Figure 9.**
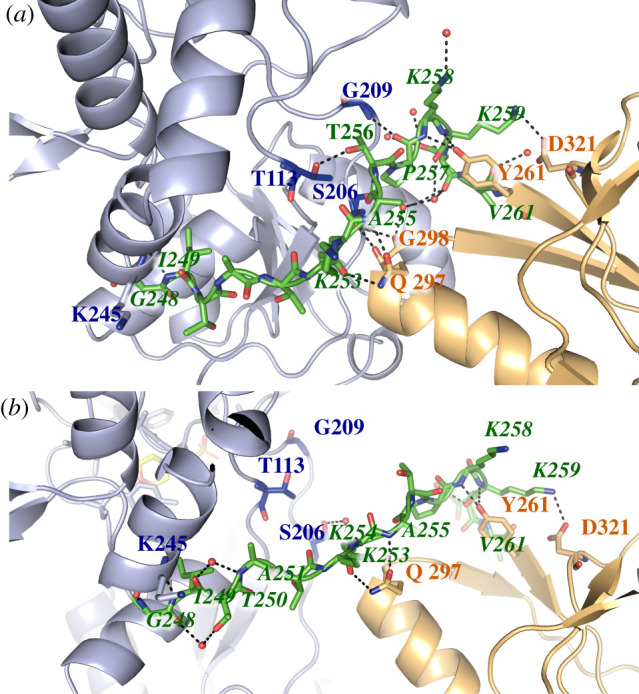
Interaction between the SBDs and their connecting linker in the crystal. Comparison between the two crystallographic monomers in the unit cell. SBD1 is depicted in blue, SBD2 in orange and linker in green. (*a*) Interactions in the compact conformation (chain A) and (*b*) in looser conformation (chain B).

We here presented a detailed study of the biochemical, biophysical and structural properties of tandem SBDs of the amino acid importer GlnPQ from *L. lactis*. We determined the crystal structure of the tandem SBDs without ligand. The packing of protein molecules in the crystal may not reflect the domain orientation of SBD1 and SBD2 in solution, but at the same time, the structures of individual SBD1 and SBD2 were in excellent agreement with the published structures of single SBDs [8]. In each tandem of the asymmetric unit (chain A/B), we observed different interactions between the domains and/or the domains and linker figure 9. Although the two observed distinct packing modes might be crystallization artefacts, it nevertheless pinpoints to an inherent mobility provided by the linker, which allows different interactions between residues of both domains (figure 9). Furthermore, chain conformation A proved to be a useful tool for the prediction of dye–protein interactions for assay design (figures 7 and 8).

To examine whether the linker does indeed impart flexibility and how the tandem arrangement might impact ligand affinity, we turned to ITC and in solution smFRET experiments. We could show that unliganded and closed liganded states of single and tandem SBDs and SBDs were identical in solution. In line with this observation, a combination of smFRET and ITC experiments revealed similar ligand affinities for the single SBDs in comparison to those in the tandem SBD1-2. We can thus conclude from this data that the interactions between the domains and the linker do not alter the domain structure and have little impact on the kinetics of conformational transitions since the binding constants are primarily determined by the closed state lifetimes of the SBDs. Our experiments, however, were not able to determine whether the orientation of the domains is free to change in solution or what might be the relevance of the different domain orientations seen in the crystal structure.

We further find that deletions and mutations of residues in the linker of wild-type SBD1-2 do not impact the biochemical properties or conformational states of the SBDs very much. Only in the extreme case of the short linker in SBD1-Δ5-SBD2, where 5 amino acids were removed, could we infer additional interactions between SBD1 and SBD2 that may explain the observed change in binding affinity in SBD1. For SBD1, the hinge between the domains is important for the Venus-Fly trap motion during substrate binding. The linker is directly positioned at the back of the hinge with which it can easily interact and thereby alter the binding characteristics. Similar behaviour has also been reported for other SBPs, such as MalE [44]. In the case of SBD1-Δ5-SBD2, the shortening of the linker might alter the orientation or rotation possibilities of SBD2 relative to SBD1, which may be reflected in altered biochemical parameters, i.e. higher affinity. In line with results from ITC experiments, we further observed in our smFRET assays, that the binding affinity of glutamine to SBD2 in SBD1-Δ5-SBD2^C^ is decreased (electronic supplementary material, figure S10). This further supports the hypothesis that SBD1 and SBD2 are no longer connected in a flexible fashion and might force SBD2 to slightly open in SBD1-Δ8-SBD2^C^ seen by a shift in mean FRET efficiency (electronic supplementary material, figure S9/S11).

To further probe inter-domain interactions and to provide a proof-of-concept experiment, we implemented solution-based PIFE–FRET [29,30] showing that it is possible to simultaneously monitor conformational states and binding affinities of one SBD while probing the proximity to the neighbouring protein domain. We have previously shown that the combination of PIFE and FRET allows probing both, short and long distances in protein–nucleic acid interactions in a single experiment. In the experiments shown here, we monitor protein–protein interactions via PIFE–FRET and provide a proof-of-concept to probe the proximity and influence of an unlabelled protein domain to a neighbouring simultaneously via smFRET.

Our data also suggest that the native coupling of both domains has no functional significance for ligand binding. We show that the tandem SBD1-ε20-SBD2 in solution has identical conformational states and binding properties as the wild-type tandem SBDs (electronic supplementary material, figure S11). In line with this, we do not find positive or negative cooperativity in the binding of ligands to one or the other SBD. Yet, the linker length is an important factor for transport activity. For instance, extensions of the linker resulted in a reduced rate of transport, presumably by increasing the delivery time for ligand from the SBDs to the TMDs of the transporter [17]. Our work clearly shows that the ligand-binding properties of each SBD are nearly unaffected by the neighbouring domain.

## 4. Material and methods

### 4.1. Preparation of reagents

Unless otherwise stated, reagents of luminescent grade were used as received. Ingredients for buffers as well as chemical compounds such as 6-hydroxy-2,5,7,8-tetramethylchro-mane-2-carboxylic acid (Trolox), dithiothreitol (DTT), EDTA, bovine serum albumin (BSA), asparagine and glutamine were purchased from Sigma-Aldrich. The radio-labelled compounds [^3^H]-asparagine and [^14^C]-glutamine were obtained from American Radiolabelled Chemicals and PerkinEllmer, respectively. Recombinant DNA reagents and primers were purchased from Merck. As calibration samples in ALEX as well as anisotropy experiments, 45 bp-long oligonucleotides (IBA, Germany) were used as received. The single-stranded DNA was labelled with Alexa Fluor 555, Alexa Fluor 647 (Thermofisher), Cy3, Cy3B (GE Healthcare) or ATTO647N (ATTO-Tec, Germany). Complementary ssDNA strands containing a donor and acceptor fluorophore were annealed [29] as FRET standards and stored at 100 µM in 20 mM Tris–HCl (pH 8.0), 500 mM NaCl, 1 mM EDTA. For these calibration experiments based on dsDNA, an imaging buffer based on PBS with 2 mM Trolox at pH 7.4 [45,46] was employed.

### 4.2. Nomenclature of GlnPQ derivatives

GlnPQ is composed of two subunits: GlnP and GlnQ. GlnP corresponds to the TMD that is N-terminally linked to SBD1 and fused to SBD2 [7]. GlnQ corresponds to the NBD. In this work, we investigate the single and linked substrate-binding domains SBD1 and SBD2. To investigate the conformation, binding-kinetics and cooperativity between both SBDs based on single-molecule FRET, we mutated single SBDs by Cys residues for labelling with fluorophores. We focussed on four distinct Cys backgrounds to probe the conformational states of each SBD and to monitor the interaction between both SBDs within the tandem. Throughout the manuscript, we omit the site-specific labelling position and denote them by superscripts. To probe conformations within SBD1, we studied A: SBD1(T159C/G87C) and B: SBD1(A136C/S221C) including their tandem mutants (e.g. SBD1(T159C/G87C)-2). In the case of SBD2, we focus on C: SBD2(T369C/S451C) as a single domain or part of the tandem. The cysteine-background of the inter-domain mutant is T: SBD1(A136C)-SBD2(T369C). We shortly refer to them as SBD1^A^, SBD1^B^, SDB2^C^, SBD1^A^-2, SBD1^B^-2, SBD1-2^C^ and SBD1^T^-2^T^. Here, SBD1-2^C^, for example, refers to the SBD-tandem with two Cys-mutations at position 369 and 451 on SBD2.

#### 4.2.1. Linker

In GlnP, both SBDs are tethered together by a flexible linker of 14 amino acids. To investigate its influence on substrate binding by SBD1 or SBD2 in the presence or absence of the second SBD, we created different Cys-containing mutants with shortened, respectively, extended amino acid sequences between both SBDs (electronic supplementary material, figure S6A). These are based on the Cys-derivatives SBD1^B^, i.e. SBD1(A136C/S221C) and SBD2^C^, i.e. SBD2(S369C/S451C), respectively. We denote them as SBD1^B^-Δ#-SBD2 and SBD1-Δ#-SBD2^C^, where Δ# denotes the number of deleted amino acids; *ε*AA denotes the number of inserted amino acids (electronic supplementary material, figure S6B). A complete list with the short and full nomenclature of all designed Cys-containing mutants with native and altered linker length is provided in electronic supplementary material, figure S6C and table S3. Mutants with shortened linker (position 248–261, electronic supplementary material, figure S6C,D) were created by removing amino acids after position 251 of the GlnP gene sequence. Mutants with extended linker (electronic supplementary material, figure S6C,D) were designed by insertion of amino acid sequence *gggsgggsgggsgggsaaql* into linker sequence between position 251 and 252. Additional point mutations such as D417F that prevent SBD1 or SBD2 from closing and substrate binding, are added at the end of the domain in brackets. SBD1-Δ5-SBD2^C^(D417F) refers to the tandem-protein with cysteine mutations at SBD1 and a point mutation at D417 in SBD2.

### 4.3. Bacterial strains, plasmids and growth conditions

The soluble SBDs were expressed in *E. coli* strain MC1061 carrying pBADnLicSBD1 and pBADnLicSBD2 and derivatives (site-directed mutants in either SBD1 or SBD2). The cells were grown in Luria–Bertani medium supplemented with 100 µg ml^−1^ of ampicillin in shake flasks. Expression was triggered at an OD_600_ of 0.5–0.6 by adding 2 × 10^−4^% w/v l-arabinose and fermentation was continued for another 2 h. Cells were harvested by centrifugation (15 min, 6000×*g*) and washed once with 100 mM KPi (pH 7.5). After resuspension in 50 mM KPi (pH 7.5), 20% glycerol and the addition of 0.1 mg ml^−1^ DNase, 1 mM MgCl_2_ and 1 mM phenylmethanesulfonyl fluoride (PMSF), the cells were disrupted by sonication. After sonication, 5 mM EDTA was added and the lysate was cleared by ultracentrifugation (90 min, 150 000 × *g*). The cell lysate was stored in aliquots at −80° after flash freezing in liquid nitrogen until used for purification.

### 4.4. Cloning and mutagenesis

The genes encoding the soluble SBDs were cloned into pBADnLIC [47], using ligation independent cloning, resulting in an N-terminal extension of the proteins with a 10-His-tag and a TEV protease site as described [47]. Site-directed mutagenesis was accomplished by the uracil excision-based cloning method, which employs pfuX7 polymerase [48]. Mutations were verified by sequence analysis (Eurofins Genomics, Germany).

### 4.5. Purification of SBD1 and SBD2 mutants

The cell lysate was thawed and mixed with 50 mM KPi (pH 8.0), 200 mM KCl, 20% glycerol (buffer A) plus 20 mM imidazole and incubated with Ni^2+^-Sepharose resin (GE Healthcare, Buckinghamshire, UK) (5.5 ml bed volume of Ni^2+^-Sepharose was used per gram of wet weight cells) for 1 h at 4°C (under mild agitation). Next, the resin was washed with 20 column volumes of buffer A supplemented with 50 mM imidazole. The His-tagged proteins were eluted in 3 column volumes of buffer A supplemented with 500 mM imidazole. Immediately after elution and concentration determination, 5 mM EDTA was added to prevent aggregation of the proteins. The His-tag was cleaved off by His-tagged-TEV protease treatment at a ratio of 1 : 40 (w/w) with respect to the purified protein, and, subsequently, the protein was dialysed against 50 mM Tris–HCl (pH 8.0), 0.5 mM EDTA plus 0.5 mM DTT overnight at 4°C. The His-tagged TEV and residual uncut protein were removed using 0.5 ml bed volume Ni^2+^-sepharose. The flow-through of the column was concentrated (Vivaspin, mwco 10 or 30 kDa for single SBD or tandem mutants, Sartorius; approx. 5 mg ml^−1^), dialysed in buffer A supplemented with 50% glycerol, split in aliquots and stored at −80°C after flash freezing. Before experiments, all proteins were further purified using size-exclusion chromatography on a Superdex-200 column (GE Healthcare, Buckinghamshire, UK). Their corresponding elution profiles are shown in electronic supplementary material, figure S12. Single SBDs elute around 17.5 ml and are discernible from tandems that feature an accelerated elution around 15.5 ml. All fractions of the eluted proteins were collected (electronic supplementary material, figure S12), and re-concentrated prior to fluorescence labelling for single-molecule experiments. The column was equilibrated in 20 mM Hepes-NaOH (pH 7.5), 150 mM NaCl. For crystallization protein was immediately used; for other experiments proteins were stored at −80°C.

### 4.6. Crystallization and structure determination

SBD1-2 crystals were grown with the hanging drop vapour diffusion method at 281 K [49]. Drops were prepared by mixing the protein (concentrated to 23 mg ml^−1^) and reservoir solution in a 1 : 1 v/v ratio. Crystals grew from a reservoir solution containing 125 mM MES (pH 6.0), 25% PEG200, 6.25% PEG3350 plus 50 mM NaF within 1–3 days. Data were collected at the beamline ID14-1, ESRF, Grenoble, France. The recorded data were processed using XDS [50] software package and revealed, that SBD1-2 crystals belong to space group C222_1_ with two molecules per asymmetric unit cell and 58% solvent content. The structure was solved by molecular replacement, using Phaser 2.1.4 as part of the CCP4 program suite [51]. To solve the unliganded structure for SBD1-2, the structure of the single domains were used (PDB 4KQP for SBD2 and 4LA9 for SBD1 [8]). The model building and corrections were carried out using the program COOT [52]. The models were refined using Phenix [53] with 5% of reflection randomly set aside to monitor the refinement progress. The overall quality of the model was assessed using the program MolProbity [54]. Final refinement statistics are shown in electronic supplementary material, table S1. The tandem SBD1-2 unliganded has been deposited to the PDB bank with the PDB ID 6H30.

### 4.7. Isothermal titration calorimetry

ITC experiments were performed as described previously [8]. Briefly, the purified SBDs were dialysed overnight against 50 mM KPi (pH 6.0), 1 mM EDTA and 1 mM NaN_3_. Isothermal titration experiments were carried out using an ITC-200 (MicroCal, GE Healthcare, Buckinghamshire, UK). For these experiments, the substrate was prepared in the dialysis buffer to minimize mixing effects. All experiments were carried out at 25°C and a mixing rate of 1000 r.p.m. The concentration of SBD1 and SBD2 and associated tandem mutants varied between 20 and 100 µM during the experiment, depending on the expected *K*_D_ of the protein under investigation. For titration experiments with asparagine typically the concentration of ligand in the syringe was 8–10× the concentration in the cell. For glutamine titrations to SBD2, the concentration in the syringe varied between 8 and 40× protein concentration. The recorded data was approximated by a one- respectively two-site binding model [55] and fitted using the nonlinear curve-fitting tool provided by ORIGIN 8 (Origin Lab Corp., Northhampton, MA) to describe the molar enthalpy change Δ*H* for protein–ligand complex formation, the stoichiometry *n* and the corresponding association constant *K*_A_. From these, we derived the dissociation constant *K*_D_ as 1/*K*_A_, and the standard free energy change of binding Δ*G* = −RT ln(*K*_A_). The molar entropy change Δ*S* was calculated from Δ*G* = Δ*H* − *T*Δ*S*. The experiments were at least repeated 3 times, if not mentioned otherwise. For analysing the glutamine binding to SBD1-Δ5-SBD2, which features two binding sites, we first determined the parameters for the single site using the conditions of asparagine binding to SBD1. Next, the analysis of the SBD1-Δ5-SBD2 mutants for the titration with glutamine was performed. Afterwards, we fixed the parameters for SBD2 for the two-site-fitting model in order to determine the binding of glutamine to SBD1.

### 4.8. Differential scanning calorimetry

To determine the proteins thermal stability, DSC experiments were performed as described previously [8]. Briefly, the purified SBDs were dialysed overnight against the DSC working buffer, i.e. 50 mM KPi (pH 7.0), 150 mM KCl, 1 mM EDTA and 1 mM NaN_3_. DSC experiments with working buffer solutions containing 4 µM of an SBD-mutant and 5 mM substrate were conducted on a VP-DSC Calorimeter (MicroCal, GE Healthcare, Buckinghamshire). The melting temperature *T*_m_ was determined by ORIGIN 8 (Origin Lab Corp, Northampton, MA).

### 4.9. Purification of Cys-containing mutants and protein labelling

Unlabelled SBD mutants with two inserted cysteines were stored at −20°C in 100 µl aliquots of 20–40 mg ml^−1^ in 50 mM KPi (pH 7.4), 50 mM KCl, 50% glycerol and 1 mM DTT. Stochastic labelling with maleimide derivatives of donor and acceptor fluorophores was carried out on approximately 5 nmol of protein with a ratio of protein : donor : acceptor = 1 : 4 : 5; SBD derivatives were labelled with two dye pairs: Alexa Fluor 555- and Alexa Fluor 647-maleimide (FRET assay) or Cy3(B)- and ATTO647N-maleimide (PIFE–FRET assay). Briefly, purified proteins were treated with DTT (10 mM; 30 min) to fully reduce oxidized cysteines. After diluting the protein sample to a DTT concentration of 1 mM, the reduced protein was bound to a Ni^2+^-Sepharose resin (GE Healthcare, UK) and washed with 10 column volumes of 50 mM KPi (pH 7.4), 50 mM KCl, 10% glycerol (buffer B). Simultaneously, the applied fluorophore stocks (50 nmol in powder) dissolved in 5 µl of water-free DMSO, were added at appropriate amounts to buffer B and immediately applied to the protein bound to the Ni^2+^-Sepharose resin (keeping the final DMSO concentration below 1%). The resin was incubated overnight and kept at 4°C (under mild agitation). After labelling, the unbound dye was removed by sequential washing with 10 column volumes of buffer B, followed by 100 column volumes of 50 mM KPI (pH 7.4), 10 mM KCl, 5% glycerol. The protein was eluted in 0.8 ml of 50 mM KPI (pH 7.4), 50 mM KCl, 5% glycerol, 500 mM imidazole and applied onto a Superdex-200 column (GE Healthcare, UK) equilibrated with 50 mM KPi (pH 7.4), 200 mM KCl.

### 4.10. Steady-state fluorescence anisotropy

Free fluorophore rotation and hence the correlation between FRET efficiency and distance were validated by steady-state anisotropy measurements. Fluorescence spectra and anisotropies *r* [56] were derived on a standard scanning spectrofluorometer (Jasco FP-8300; 20 nm exc. and em. width; 8 s integration time) and calculated at the emission maxima of the fluorophores (e.g. *λ*_em_ = 570 nm for Cy3(B), and *λ*_em_ = 660 nm for ATTO647N) according to the relationship *r* = (*I*_VV_ – *GI*_VH_)/(*I*_VV_ + 2*GI*_VH_). The excitation wavelengths at *λ*_ex_ = 532 nm resp. *λ*_ex_ = 640 nm were chosen according to the laser lines employed for µs-ALEX spectroscopy. *I*_VV_ and *I*_VH_ describe the emission components relative to the vertical (V) or horizontal (H) orientation of the excitation and emission polarizer. The sensitivity of the spectrometer for different polarizations was corrected using horizontal excitation to obtain *G* = *I*_HV_/*I*_HH_. Typical *G*-values for Cy3(B) and ATTO647N were 0.64 ± 0.03 and 0.45 ± 0.03. *G*-values for Alexa Fluor 555 and Alexa Fluor 647 were determined to be 1.8–1.9 [19]. We analysed the anisotropy of double-labelled protein mutants and DNA samples in a concentration range of about approximately 100 nM. The determined anisotropy values are summarized in electronic supplementary material, table S4.

### 4.11. Sample preparation for single-molecule experiments

μs-ALEX-experiments were carried out at 25–50 pM of double-labelled protein or DNA in buffer containing 50 mM KPi (pH 7.4), 150 mM KCl, 1 mM Trolox and 10 mM MEA. ALEX titration experiments on GlnPQ, i.e. a chosen SBD or tandem mutant in presence of varying ligand concentrations, were completed in one continuous experiment. To monitor and detect possible changes in the experimental settings, every set for ALEX experiments on SBDs was complemented by an experiment of dsDNA FRET standard [18] of 45 bp length (data not shown). The dsDNA was labelled either with Cy3(B) and ATTO647N or Alexa Fluor 555 and Alexa 647 in 18 and 23 bp distance depending on the labelling scheme of the SBDs.

### 4.12. Single-molecule FRET and ALEX spectroscopy

μs-ALEX-experiments were carried out at room temperature (22°C) on a custom-built confocal microscope [18,29]. In brief, ALEX between 532 and 640 nm was employed with an alternation period of 50 µs, coupled into a confocal microscope, a 60× objective with NA = 1.35 (Olympus, UPLSAPO 60XO) focused the excitation light to a diffraction-limited spot 20 µm into the solution. The excitation intensity amounted to 60 µW at 532 nm (≈30 kW cm^−2^) and 25 µW at 640 nm (≈25 kW cm^−2^). Fluorescence emission was collected and spectrally separated onto two APDs (τ-spad, Picoquant, Germany) with appropriate filters (donor channel: HC582/75; acceptor channel: Edge Basic 647LP; AHF Analysentechnik, Germany). The signal was recorded using a custom-written LabView program.

### 4.13. ALEX data extraction and analysis

After data acquisition, the recorded fluorescence emission was analysed and processed using custom-made scripts in Python. Fluorescence photons arriving at the two detection channels (donor detection channel: *D*_em_; acceptor detection channel: *A*_em_) were assigned to either donor- or acceptor-based excitation based on their photon arrival time. From this, three photon streams were extracted from the data corresponding to donor-based donor emission *F*(DD), donor-based acceptor emission *F*(DA) and acceptor-based acceptor emission *F*(AA). For each molecule diffusing through the confocal volume, fluorophore stoichiometries *S* and apparent FRET efficiencies E* were calculated for each fluorescent burst above a certain threshold yielding a two-dimensional histogram [36,37]. Uncorrected (apparent) FRET efficiency E* monitors the proximity between the two fluorophores and is calculated according to

4.1 $$E^{*}=\frac{F(\mathrm{DA})}{F(\mathrm{DD})+F(\mathrm{DA})}.$$

*S* is defined as the ratio between the overall green fluorescence intensity over the total green and red fluorescence intensity and describes the ratio of donor-to-acceptor fluorophores in the sample:

4.2 $$S=\frac{F(\mathrm{DD})+F(\mathrm{DA})}{F(\mathrm{DD})+F(\mathrm{DA})+F(\mathrm{AA})}.$$

Using published procedures to identify bursts corresponding to single molecules [57], we obtained bursts characterized by three parameters (*M*, *T* and *L*). A fluorescent signal is considered a burst provided it meets the following criteria: a total of *L* photons, having *M* neighbouring photons within a time interval of *T* microseconds. For all data presented in this study, an all photon burst search [57,58] using parameters *M* = 15, *T* = 500 µs and *L* = 25 was applied; additional thresholding removed spurious changes in fluorescence intensity and selected for intense single-molecule bursts (all channels greater than 150 photons). After binning the detected bursts into a 2D E*/*S* histogram, sub-populations were separated according to their *S*-values. E*- and *S*-distributions were fitted using a 2D Gaussian function, yielding the mean values $\mu_{i}$ of the distribution and an associated standard deviation.

### 4.14. Population assignment

To correct individual populations, i.e. *apo*-protein state and closed liganded within one 2D ALEX histogram, every burst needs to be assigned to a particular population. This can be achieved via cluster analysis methods or probability distribution analysis [59]. In our implementation, every population in the uncorrected 2D histogram is first fitted with a covariant bivariate Gaussian function

4.3 $$f_{i}(E,S)=A\;\exp\left\{-\frac{1}{2(1-\rho^{2})}\cdot \left[{\left(\frac{E-\mu_{E}}{w_{E}}\right)}^{2}-2\rho \cdot \left(\frac{E-\mu_{E}}{w_{E}}\right)\cdot \left(\frac{S-\mu_{S}}{w_{S}}\right)+{\left(\frac{S-\mu_{S}}{w_{S}}\right)}^{2}\right]\right\},$$

where the population is described by an amplitude *A*, its mean values $\mu_{i}$ and standard deviations $w_{i}$ in FRET *E* and stoichiometry *S*. ρ denotes the correlation matrix between *E* and *S*. We express the probability *p* that a given burst in the 2D histogram belongs to a population *i* by

4.4 $$p_{i}(E,S)=\;\frac{\;f_{i}(E,S)}{\sum_{\;j=1}^{n}f_{j}(E,S)}.$$

### 4.15. Titration experiments

To investigate the binding affinity of the labelled SBDs, and hence the transition between open unliganded and closed liganded conformation, titrations in ALEX experiments in the presence of high-affinity ligands were carried out. The two-dimensional *E*- and *S*-distributions were fitted using 2D Gaussian functions, yielding the mean values $\mu_{i}\;$of the distribution and an associated standard deviation $w_{i}$. At first, the histograms of *apo*-protein and protein at fully saturating substrate concentration were investigated. Their projections in *E* represent the FRET distributions of the open unliganded and closed liganded state, respectively. Subsequently, these two distributions were employed to fit the titration data at intermediate substrate concentration via a Hill model with fixed *V*_max_ value using ORIGIN 8 (Origin Lab Corp, Northampton, MA). The fractional occupancy of the high FRET Gaussian as a function of substrate concentration was fitted afterwards with a one-side-binding model, which allowed calculation of *B*_max_ (maximal fraction of closed state) and *K*_D_ (dissociation constant).

### 4.16. PIFE data extraction and analysis

To monitor the presence of the second SBD and the intra-domain distance within the tandem by PIFE, ALEX experiments in presence of high-affinity ligands were carried out, i.e. ALEX spectroscopy on SBDs labelled with Cy3/ATTO647N-maleimide were carried without in absence and presence of a saturating ligand. The two-dimensional E*- and S*-distributions were fitted using 2D Gaussian functions (equation 4.3), yielding the mean values $\mu_{i}\;$of the distribution and an associated standard deviation $w_{i}$. The shift in brightness of Cy3 in presence of the second SBD is seen as a shift in stoichiometry between the single SBD and as part of a tandem. Therefore, at first, the histogram of the single SDB as *apo*-protein and fully saturating substrate concentration were investigated. Their stoichiometry values are taken as reference. We report the change in stoichiometry ΔS* as a function of linker length, and hence the distance between both SBDs.

### 4.17. Burst variance analysis

To reveal any static and/or dynamic heterogeneity in single-molecule ALEX data, we employed BVA [39]. Here, we compare the expected shot-noise limited standard deviation $\sigma_{E^{*}}^{2}$ for a given mean FRET efficiency $E^{*}$ against the actual standard deviation for individual molecules. The expected standard deviation $\sigma_{E^{*}}^{2}\;$due to shot-noise depends only on photon statistics and reads as

4.5 $$\sigma_{E^{*}}^{2}=\;\sqrt{\frac{E^{*}(1-E^{*})}{N_{\mathrm{DD}+\mathrm{DA}}}},$$

where $N_{\mathrm{DD}+\mathrm{DA}}$ is the average number of photons per burst emitted by the double-labelled molecule after green excitation. Similar burst selection criteria as described above where used: all channels greater than 250 photons and only burst within the stoichiometry range from 0.3 to 0.6 were used.

### 4.18. Structural modelling and accessible volume calculation

To compare distances within the obtained crystal structure with results determined by FRET, we carried out structural modelling and AV calculations. We visualize the individual and linked SBDs, as well as the position at which both fluorophores are stochastically attached, based on four different crystal structures—SBD1 in the *apo*-state (PDB [8] 4LA9) and in presence of asparagine (PDB 6FXG), SBD2 in the *apo*-state (PDB [8] 4KR5) and presence of glutamine (PDB [8] 4KQP)—in comparison to the published structure of the linked SBDs. We loaded the respective pdb files in PyMOL [60] and removed co-crystallized items, like ligands and proteins. Next, we determine the ID of each CB atom to which the fluorophores (i.e. Cy3(B) resp. Alexa Fluor 555 and ATTO647N resp. Alexa Fluor 647) are attached via cysteine-maleimide click-chemistry. With this knowledge, we determined the AV and expected distances between the dyes [43] on the protein complex in the unliganded and unliganded case. The dyes were attached to the *C*_β_ atom of the corresponding amino acids and simulated as C2 maleimide derivative with parameters as specified in the FPS software manual [43]. Afterwards, we use PyMOL to compare and display the determined AVs and distance within the crystal structures.

## Data accessibility

Data sets are available from Zenodo [61].
